# Échecs thérapeutiques chez les enfants infectés par le VIH en suivi de routine dans un contexte à ressources limitées au Cameroun

**DOI:** 10.11604/pamj.2013.15.80.2754

**Published:** 2013-06-30

**Authors:** Calixte Ida Penda, Francine Same Bebey, Danielle Kedy Mangamba, Else Carole Eboumbou Moukoko, Victoria Ngwa, Nicaise Makouet, Anne-Cécile Bissek, Blaise Dupont Minkemdefo, Ekoe Tetanye, Paul Koki Ndombo

**Affiliations:** 1 Faculté de Médecine et des Sciences Pharmaceutiques, Université de Douala, Cameroun; 2 Hôpital Laquintinie de Douala, Cameroun; 3 Groupe technique régional du littoral du comité de lutte contre le sida; 4 Faculté des Sciences Biomédicales, Université de Yaoundé 1, Cameroun

**Keywords:** VIH, enfants, échec thérapeutique, HIV, children, treatment failure

## Abstract

**Introduction:**

L'objectif de cette étude était de déterminer les facteurs associés aux échecs thérapeutiques chez les enfants infectés par le VIH à l'Hôpital Laquintinie de Douala.

**Méthodes:**

Une étude transversale rétrospective a été menée sur une période de 5 mois en 2010, recrutant 222 enfants âgés de 1 à 18 ans et sous TARV depuis au moins 24 semaines. Les données sociodémographiques, cliniques, biologiques et de l'observance thérapeutique des patients ont été collectés à partir des dossiers des patients, et analysées avec le logiciel SPSS (version 16).

**Résultats:**

39 (17,6%) des enfants étaient en échec thérapeutique (délai moyen de survenue 26,8 mois) et 73,4% d'entre eux sont passés en seconde ligne. Les garçons avaient en moyenne un risque 5 fois plus élevé de faire un échec thérapeutique que les filles (OR=3,9; p=0,035). 94,4% des enfants suivis avaient un faible taux de CD4 à l'initiation (‘ 25%) associé au risque élevé d’échec thérapeutique (OR=5,2; p=0,007). Les enfants issus de famille monoparentale représentaient près de la moitié des cas d’échecs thérapeutiques. Sur 39 cas en échec thérapeutique, 41% des enfants étaient des orphelins. Parmi les enfants sous TARV, 46% prenaient leur trithérapie sous forme de médicaments séparés parmi lesquels 52,1% étaient en échec thérapeutique.

**Conclusion:**

Les échecs thérapeutiques et le passage en seconde ligne dépendaient du contexte familial des enfants, de leur statut immunologique à l'initiation du traitement, de leur sexe et de la forme galénique du TARV.

## Introduction

En 2010, on estimait à 3,4 millions les enfants de moins de 15 ans vivant avec le VIH avec 390 000 nouvelles infections [1, 2]. Au Cameroun, ce nombre étaient estimé à 48 736 parmi lesquels 31 969 (65,6%) enfants éligibles aux antirétroviraux et 4 440 (13,8%) mis sous traitement antirétroviral (TARV) en 2010 [3, 4]. L'accès au TARV est effectif depuis 2002 au Centre de traitement agrée (CTA) de Hôpital Laquintinie de Douala (HLD) et gratuit depuis 2007. En dehors de la charge virale, les bilans de suivi biologique de l'enfant infecté par le VIH sont subventionnés au Cameroun mais non disponibles de façon continue. Par ailleurs, la participation au coût des examens reste néanmoins chère pour les familles. On parle de l’échec thérapeutique quand l'enfant a reçu un TARV sans succès après une période d'au moins 24 semaines. Plusieurs facteurs peuvent contribuer à cet échec: une observance insuffisante, une rupture de stock de médicaments et une existence préalable d'une résistance. La numération des CD4 basse (6,0 log), la malnutrition aigue sévère sont des facteurs prédictifs de l’échec de traitement à l'initiation qui augmentent le risque de mortalité [5, 6]. Les différences génétiques dans le métabolisme des ARV peuvent aussi jouer un rôle important [7]. Lors du TARV, la découverte d'un évènement clinique nouveau ou récurrent du stade clinique III ou IV peut indiquer la progression de l'infection à VIH et l’échec devrait être envisagé si l'observance du traitement a été satisfaisante chez l'enfant [8, 9]. L'observance est évaluée et jugée optimale en dehors de toute infection intercurrente, de toute pathologie grave traitée et guérie, ou de tout diagnostic du syndrome inflammatoire de reconstitution immunitaire [2, 9]. L'observance du traitement de l'enfant dépend du tuteur. La préparation, l'adhésion des parents au respect du traitement prescrit et de la disponibilité des formes galéniques adaptées à l’âge sont les facteurs clé pour l'obtention d'une bonne observance [5, 10]. Les enfants sont souvent pris en charge tardivement en Afrique, comme le révèle l’étude Ouest-Africaine de 2 400 enfants infectés par le VIH où l’âge moyen au début du TARV était de 4,9 ans [11]. Ce retard de prise en charge pourrait avoir un impact sur les échecs du fait des comorbidités et des multithérapies. Une étude réalisée en Afrique du Sud suggère qu'un début précoce du TARV permet de réduire de façon spectaculaire le risque de mortalité de 76% et la progression de l'infection à VIH de 75% chez le nourrisson [12, 13]. Le suivi des indicateurs d'alerte précoce (IAP) de résistance du VIH aux antirétroviraux (R-ARV) sur les sites permet de prévenir lesdites résistances. Ces IAP sont des facteurs qui peuvent être associés à l’émergence de R-ARV [2, 10]. Notre étude avait pour but de déterminer les facteurs associés à l’échec thérapeutique, d'apprécier l'apport des méthodes diagnostiques ainsi que ceux des indicateurs d'alerte précoce chez les enfants infectés par le VIH sous TARV au CTA de l'HLD.

## Méthodes

### Site d’étude et méthodologie

Il s'agit d'une étude transversale rétrospective menée au CTA de l'HLD pendant 5 mois en 2010 sur un échantillonnage simple et consécutif des dossiers complets des enfants âgés de 1 à 18 ans infectés par le VIH et sous TARV depuis au moins 24 semaines. L′âge, le sexe, le statut familial, le stade clinique de l'OMS à l'initiation du TARV, la forme du médicament, l'observance thérapeutique, les données biologiques (immunologiques, virologiques), le type de traitement et les comorbidités liés au VIH survenus au cours de la durée du traitement ont été collectés et enregistrés sous forme «adoc» pour chaque enfant. La confidentialité des données a été assurée par le remplacement des noms par des codes. Un consentement éclairé du parent ou du tuteur légal de chaque enfant a été obtenu. Le protocole d’étude a été approuvé par le Comité d'Ethique et de recherche de l'HLD.

### Prise en charge des enfants VIH et diagnostic de l’échec thérapeutique

Chez les nouveaux-nés, le diagnostic du VIH a été fait par PCR à partir de la sixième semaine et avant 9 mois. En cas de PCR positive, l'enfant était mis sous traitement précoce. Pour les enfants âgés de 9 à 15 mois, la sérologie était faite à leur arrivée et en cas de séropositivité, une PCR de confirmation était réalisée. Au delà de 15 mois, seule une sérologie a été faite. Les nourrissons de moins de 24 mois avec une PCR positive était mis sous TARV de manière systematique et le bilan prethérapeutique était demandé. Pour les enfants âgés de 24 mois et plus, un bilan prétherapeutique était demandé quand la sérologie ou la PCR était positive et avant tout traitement. Le TARV de 1^ère^ ligne dépendait de 3 critères: le taux de CD4, le stade clinique OMS de l'enfant et la prise des ARV par la mère dans le cadre de la prévention de la transmission mère-enfant (PTME) du VIH. Les traitements utilisés en 1^ère^ et 2^ème^ ligne sont resumés dans le Tableau 1.

**Tableau 1:** Régimes thérapeutiques des ARV utilisés à l'Hôpital Laquintinie de Douala^*^

Groupe d'enfants	Traitement de première ligne	Traitement de seconde ligne
Enfant non exposé à la NVP	NVP+(D4T ou AZT) + 3TC	Lop/r + Videx + Abacavir
Enfant exposé à la NVP	Lop/r +(D4T ou AZT) + 3TC	EFV + Videx + AZT
Enfant dont l’ exposotion à la NVP n'est pas connue	NVP + (D4T ou AZT) + 3TC	Lop/r + Videx + Abacavir
Enfant de 3 ans et plus	NVPou EFV + (D4T ou AZT) + 3TC	Lop/r + Videx + Abacavir

* NVP, Névirapine ; D4T, le Zérit; AZT, Zidovudine; 3TC, Lamivir; Lop/r, Lopinavir boosté de Ritonavir; EFV, Efavirenz

**Figure 1:**
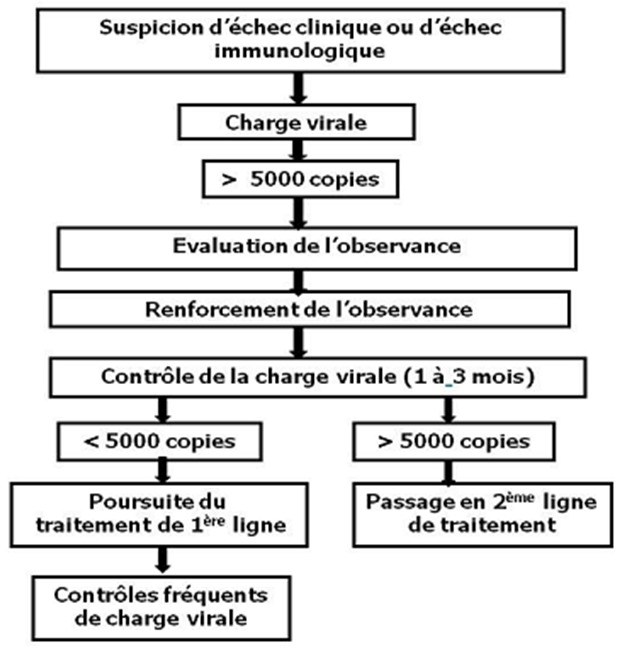
Algorithme utilisé pour la prise en charge des échecs thérapeutiques selon les recommandations de l'OMS

Selon les critères de l'OMS, l’échec thérapeutique était défini par l’échec clinique, immunologique et/ou virologique. La prise en charge de l’échec thérapeutique était basée sur le renforcement de l’éducation thérapeutique et le changement thérapeutique selon l'algorithme de l'OMS (Figure 1). Les IAP permettant de prévenir la survenue des résistances aux ARV sur site étaient basés sur les variables comme la prescription, l'adhésion, l'observance, et l'histoire thérapeutique (approvisionnement, durée, efficacité, et tolérance). Le suivi des IAP s'est faite par (1) le recueil de la pratique de la prescription des ARV (IAP 1P), (2) le nombre de patients perdus de vue au cours des 12 premiers mois du TARV (IAP 2), (3) le pourcentage de patients encore sous première ligne de TARV après 12 mois (IAP 3P), (4) le nombre de patients ayant retiré les ARV dans les délais à la pharmacie (IAP 4), (5) le nombre de patient ayant respecté les rendez-vous de consultation de suivi du TARV (IAP 5) et (6) le pourcentage de mois sans rupture de stock de médicament au cours de l'année désignée (IAP 6).

### Analyse statistique

La description simple de l’échantillon a été possible grâce au calcul des proportions ou des moyennes selon que la variable soit catégorielle ou numérique. Les tests du Chi2 et de Fisher (lorsque celui du Chi2 était inapproprié) ont été utilisés pour déterminer la signification entre les variables catégorielles. Seules les variables associées au pronostic dans le modèle univarié ont été analysées dans un modèle de régression logistique multivariée, tenant compte ainsi de toutes les facteurs confondants. Afin d’évaluer les indicateurs de risque et la liaison entre un facteur d'exposition et la survenue de l’échec thérapeutique, l'Odds Ratio (OR) a été calculé. Le risque d'erreur était fixé à 5% avec un intervalle de confiance (IC) de 95%. Toutes les analyses ont été faites en utilisant le logiciel SPSS (version 16.0 pour Windows).

## Résultats

### Population d’étude, profile démographique

Sur 303 dossiers étudiés, 81dossiers d'enfants ont été exclus parmi lesquels 27 concernaient les enfants suivis sans TARV et 54 avaient une durée du traitement inférieure à 24 semaines. Les caractéristiques sociodémographiques, thérapeutiques et cliniques des 222 enfants inclus dans l’étude sont présentées dans le Tableau 3. L’âge des enfants était compris entre 0,5 et 18 ans avec un âge moyen de 8,23 ans (+/- 3,72). Quatre vingt quatorze enfants (42,3%) étaient des filles et le sexe ratio F/M était de 0,7.

### Diagnostic de l'infection virale et paramètres cliniques

Le VIH de type 1 du groupe M était responsable à 99,5% de l'infection. A l'initiation du traitement, 84 (37,8%) des enfants étaient au stade clinique III et 75 (33,8%) au stade IV OMS du SIDA, 27,5% au stade II et 0,9% était au stade I. Le taux de CD4 des enfants inclus variait de 1 à 60% à l'initiation du traitement avec une moyenne de 16,4% (OR=5,1; 95%IC=1,3-23,6; p=0,007).

### Traitement sous ARV

Tous les patients ont reçu un TARV de première ligne à l'initiation: 54% sous forme de combinaison à dose fixe et 46% sous forme de trois de médicaments séparés (sirop et/ ou comprimés). La durée moyenne du TARV était 1,2 fois plus élevée chez les enfants en échec thérapeutique que chez les enfants sans échec et cette différence était significative (p=0,05) (Tableau 3). La proportion d'enfants sous TARV ayant fait la numération des CD4 au 6^ème^, 12^ème^ et au 24^ème^ mois variaient entre 62 à 67%. La mère était directement responsable de l'administration du traitement chez 67,1% des 222 patients.

### Echec thérapeutique

Parmi les 222 enfants inclus, 39 (17,6%) étaient en échec thérapeutique alors que 82,4% avaient eu une bonne évolution (Tableau 3). L’âge des enfants en échec thérapeutique était compris entre 3 et 18 ans (moyenne 9,5 ans). 71,8% des garçons présentaient un échec thérapeutique par rapport aux filles (28,2%) (p< 0,035). Parmi les enfants en échec thérapeutique, 41% (16/39) étaient des orphelins. La proportion d'enfants en échec thérapeutique ayant fait la numération des CD4 à M6, M12, M24 étaient en moyenne de 76%. Près de la moitié (46%) des enfants ont effectué une charge virale et 3% étaient en échec virologique à M6, 8,8% à M12 et 9,8% à M24.

**Figure 2:**
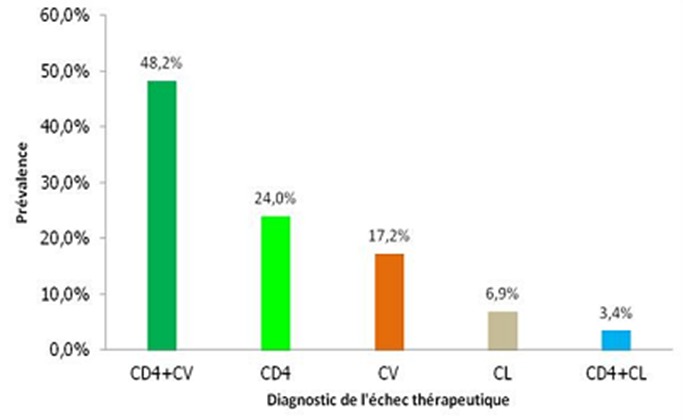
Diagnostic de l’échec thérapeutique

Le diagnostic de l’échec thérapeutique était posé sur la base de: la numération des CD4 et de la charge virale pour 48.2%; chez 24,1% sur la numération des CD4 seuls; 17,2% sur la charge virale seule, 6,9% sur la survenue d’évènements cliniques classant stade III ou IV au cours du traitement ARV et 3,6% sur la clinique et la numération des CD4 (Figure 2). A l'initiation du traitement des enfants en échec, 43,6% étaient au stade IV; 35,9% au stade III; 20,5% au stade clinique II de l'OMS et aucun enfant du stade I. Concernant leurs CD4 initiaux, 94,9% avaient des CD4 inférieure ou égale à 25%.

**Figure 3:**
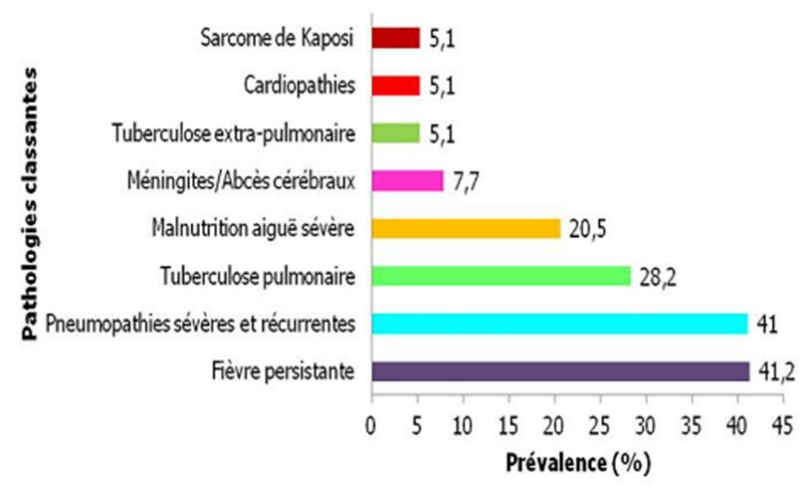
Evénements Cliniques de stades III et IV survenus au cours du traitement

**Figure 4:**
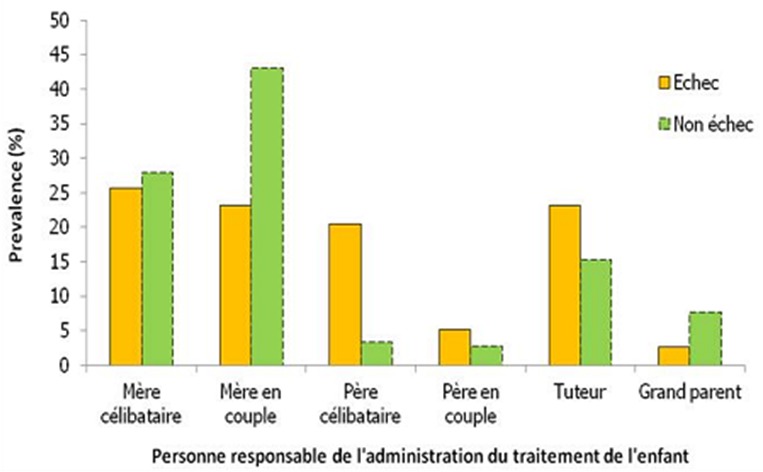
Distribution de l’échec thérapeutique en fonction de la personne responsable de l'administration du traitement à domicile de l'enfant “Caregivers”

En ce qui concerne les comorbidités liées au VIH, la fièvre au long cours et les infections pulmonaires à répétitions représentaient les évènements cliniques classant du stade III les plus fréquemment identifiés au cours du traitement des enfants en échec thérapeutique soit respectivement 41,2% et 41% (Figure 3). Près de 70% des enfants en échec présentaient des dermatoses au cours de leur traitement. Le prurigo était la dermatose le plus représenté (37%). Le traitement étaient administré par les mères chez 48,7% des enfants en échec thérapeutique (Figure 4). Parmi les mères, 25,6% étaient célibataires et 23,1% vivaient en couple. Les tuteurs et les pères célibataires représentaient 43,6%; les pères en couple et les grands-parents respectivement 5,1% et 2,6%. La durée du traitement ARV des enfants en échec thérapeutique variait de 12 à 100 mois, avec une moyenne de 51,5 mois (Tableau 3). Le délai moyen de survenue des échecs était de 26,8 mois. Parmi les patients en échec 56,4% (22/39) ont respecté leur rendez-vous aux consultations de routine pendant les 12 premiers mois de traitement et 53, 8% (21/39) pendant toute la durée de leur traitement. Sur 39 «caregivers» des enfants, 16 (41%) se sont présentés régulièrement à la pharmacie pour l'approvisionnement en ARV de leur enfant durant les 12 premiers mois, 7,7% (3/39) ont été retirés les ARV de leur enfant dans les délais pendant la durée entière du traitement. Parmi les enfants en échec thérapeutique, nous avons enregistré 2 perdus de vue (5,1%). Au cours du suivi, les périodes durant lesquelles les «caregivers» des enfants en échec ne sont pas venus chercher leur traitement ARV variaient de 0 à 41 mois (moyenne: 11,15 mois; écart-type: 9,42 mois). Chez 2 enfants en échec thérapeutique, les ARV ont été remplacés pour rupture de leur traitement ARV à la pharmacie.

Pour tous les cas d’échec thérapeutique rencontrés, 29 (73,4%) sont passés au traitement de seconde ligne selon l'algorithme de l'OMS utilisé.

### Les indicateurs pédiatriques d'alerte précoces (IAP) des resistances aux ARV(R-ARV)

- IAP 1P: Tous les patients recevaient un traitement ARV de première ligne à l'initiation parmi lesquels 54% ont eu leur trithérapie sous formes de combinaisons à dose fixe et 46% sous formes trois médicaments. Parmi les enfants en échec thérapeutique, 52 % ont reçu leur traitement sous formes de trois médicaments
- IAP 2: 17(7,6%) patients étaient perdus de vue à 12 mois et les visites à domicile ont permis de ramener 3 d'entre eux
- IAP 3P: Parmi les 222 enfants, 94,2% étaient encore sous TARV de première ligne après 12 mois de traitement
- IAP 4: Parmi les 222 enfants, 80,6% des enfants sont venus chercher le TARV dans les délais durant les 12 premiers mois. Pour les enfants en échec, seuls 41% ont été régulièrement approvisionnés en ARV durant les 12 premiers mois
- IAP 5: Près d'1 enfant sur 2 a respecté son rendez-vous de consultation dans le cadre du traitement antirétroviral
- IAP 6: Rupture d'une molécule en 2007 pendant trois mois, soit 75 % de mois sans aucune rupture de stock (Tableau 2)

**Tableau 2:** Indicateurs d'alerte précoce de résistance du VIH aux antirétroviraux de l'OMS sur le site pédiatrique de l'HLD

Désignation	Indicateurs d'alerte précoce	Objectif à atteindre selon OMS	Enfants non échec	Enfants en échec thérapeutique
**IAP 1: Habitudes de prescription de TARV**	Enfants ayant eu une prescription de TARV de 1^ère^ intention approprié et dont le TARV a été retiré à la pharmacie par le «caregiver*»	100%	100%	100%
	Prescription de TARV sous forme de combinaison à dose fixe		47,9%	54%
	Prescription de TARV sous forme de molécules libres		52,1%	46%
**IAP 2: Perdus de vue**	Pourcentage d'enfants perdus de vue dans les 12 mois après le début de leur TAR	< 20%	7,6%	5,1%
**IAP 3: Taux de rétention des patients sous TAR de 1^ère^ intention**	Enfants ayant débuté un TARV sur le site et qui prenaient toujours le schéma thérapeutique de TARV initial approprié après 12 mois	> 70%	94,2%	5,8%
**IAP 4: Retrait des ARV dans les délais**	Enfants dont le «caregiver » a retiré dans les délais tous les ARV prescrits pendant les 12 premiers mois	> 90%	93%	41%
	Enfants dont le «caregiver » a retiré dans les délais tous les ARV prescrits pendant toute la durée du traitement		90%	7%
**IAP 5: Respect des rendez-vous pour les consultations de TARV**	Enfants ayant honoré tous leurs rendez-vous de consultation dans les délais pendant les 12 premiers mois du TARV	> 80%	90%	56.4%
	Enfants ayant honoré tous leurs rendez-vous de consultation dans les délais pendant toute la durée du TARV		82%	53.8%
IAP 6: Pénuries et ruptures de stock d'ARV**	mois sans rupture de stock d'ARV pédiatriques au cours d'une période désignée de 12 mois	> 100%	75%	75%

* Personnes responsables de l'administration du traitement à l'enfant ;

** Rupture pendant 3 mois d'une molécule d'ARV entrant dans la composition de la trithérapie D4Ten 2007 et en EFV 2009

**Tableau 3:** Facteurs associés à l’échec thérapeutique des enfants infectés par le VIH à l'hôpital Laquintinie au Cameroun

	Echec Thérapeutique	Non Echec Thérapeutique	Total	OR (IC 95%)	p
	N=39	N=183	N=222		
Age Moyen (min-max), ans	9,5 (3-18)	8,0 (0,5-18)	8,3 (0,5-18)	-	0,05
Garçons, n(%)	28 (71,8)	100 (54,6)	128 (57,66)	3,99 (0.20- 0.98)	0,035
					
**CD4 initiation (n=213)**					
≤25%	34 (94,4)	134 (75,7)	168	5,26 (1,28-24,43)	0,007
>25%	2 (5,6)	43 (24,3)	45	-	
					
**Stade clinique OMS à l'initiation**					
1	0 (0)	2 (1,1)	2 (0,9)	-	NS
2	8 (20,5)	53 (29,0)	61 (27,5)	-	
3	14 (35,9)	70 (38,3)	84 (37,8)	-	
4	17 (43,6)	58 (31,7)	75 (33,8)	-	
Durée moyenne du traitement (SD), mois	51,5 (25,2)	42,8 (21,6)	44,3 (22,5)	-	0,05
					
**Statut d'orphelin, n(%)**	14 (35,9)	69 (37,7)	83 (37,4)	-	NS
orphelin de mère	10 (25,6)	33 (18,0)	43 (19,4)	-	
orphelin de père	1 (2,6)	18 (9,8)	19 (8,6)	-	
orphelin de mère et de père	3 (7,7)	18 (9,8)	21 (9,5)	-	

## Discussion

La standardisation des dossiers et le respect des procédures dans cette structure nous ont permis de collecter toutes nos données. La population étudiée était constituée de 222 enfants, effectif, proche de celui de Kisito H. et col avec 259 enfants [14] et inférieur à celui de Zanoni et col en 2012 avec 880 enfants [15]. Dans notre étude, la population était composée en majorité de garçons (57,7%), distribution différente de celle observée dans l’étude de Lubega I. et col [16] avec 53% de filles. L’âge moyen des enfants dans notre série était de 8,23 ans, valeur proche de celle retrouvée (8,9 ans) dans la série de Zoufaly A.et col [17] mais supérieure à celle retrouvée dans la série de Isaakidis P. et col en 2009 et qui était de 6 ans [18]. La durée moyenne du traitement ARV dans notre population d’étude variait de 6 à 117 mois (moyenne 44,3 mois). Cette durée était inférieure à celle retrouvée dans la série de Lubega I. et coll (moyenne de 48 mois) [16] et supérieure à celle retrouvée dans la série de Isaakidis P. et coll. (moyenne de 36,2 mois) [18]. La durée du traitement ARV des enfants en situation d’échec variait de 12 à 100 mois (moyenne 51,5 mois). Mecikovsky D. et col avaient trouvé une durée moyenne de traitement ARV de 62,5 mois dans leur population d’échec thérapeutique et avaient suggéré que la résistance aux ARV (R-ARV) était liée à la durée de l'exposition aux ARV [19].

Sur 222 enfants traités depuis au moins 24 semaines, 17,6% ont été en échec thérapeutique dans notre population. Isaakidis P. et col avait trouvé des résultats similaires avec 16% d’échecs sur 158 [18]. Ce taux d’échec est supérieur à celui retrouvé dans la série de Davies M. A. en 2009 avec 310 (5,6%) cas d’échec sur 5484 [20]. A l'initiation du traitement, 71,5% des enfants dans notre série étaient au stade III et stade IV de l'OMS. Nos résultats sont similaires à ceux de Davies M.A. et col trouvé en 2009 avec 75% des enfants au stade III et IV de l'OMS à l'initiation du traitement ARV [20]. Le taux moyen de CD4 à l'initiation du traitement était de 16,8% parmi lesquels 94,4% avaient un taux de CD4 inférieur à 25%. Ces résultats comparables à ceux trouvés par Lubega I. et col qui trouvait un taux moyen de 17% [16]. Dans notre étude, lorsque le traitement avaient été initié précocement au stade clinique I, le taux d’échec était nul, tandis que 78,6% des échecs ont été observés chez les enfants initiés au stade clinique IV avec un taux moyen de CD4 initial de 12,5%. Ces résultats suggèrent une relation entre le stade clinique avancé à l'initiation thérapeutique et l’échec du traitement comme l'a observé Mecikovsky D.et col dans une autre étude [19]. Sur 39 cas d’échecs thérapeutiques, 41% des enfants étaient orphelins, parmi lesquelles les orphelins de mère étaient trois fois plus nombreux. Nos résultats sont différents de ceux de Isaakidis P. et col qui ne trouvaient aucune relation entre le statut d'orphelin et l’échec thérapeutique [18]. Dans notre série, le diagnostic de l’échec thérapeutique était basé sur la clinique, la numération des CD4 et le dosage de la charge virale. Le dosage de la charge virale était recommandé une fois par an pour les enfants sous traitement. Seuls les enfants ayant une évolution clinique ou immunologique défavorable avaient bénéficié du dosage de la charge virale anticipée à M6. Même si ces résultats montrent l'efficacité du dosage de la charge virale dans le diagnostic de l’échec thérapeutique, ils mettent en évidence d'une part la disparité de la gratuité du dosage de la charge virale et d'autre part, l'inaccessibilité financière des familles au bilan biologique du fait du contexte socio-économique. En effet, la majorité des familles ne font le dosage de la charge virale que lorsqu'il leur est proposé gratuitement. Le respect du calendrier de visite était médiocre chez les enfants en échec par rapport à la population étudiée. En effet, sur 39 enfants, plus de la moitié (56,4%) ont respecté leur rendez-vous aux consultations de routine pendant les 12 premiers mois de traitement, ce pourcentage continuait à diminuer pendant toute la durée de leur traitement pour atteindre 53,8%. Dans la population d'enfants, 7,6% (17/222) patients étaient perdus de vue parmi lesquels 5,1% (2/39) étaient en échecs alors que Fenner L. et col avait un taux de perdus de vue environ 2 fois supérieur (13,3%) à celui de notre population [21]. 94,2% des enfants sous TARV de première ligne à l'initiation y étaient encore après 12 mois de traitement. L’échec thérapeutique survenait plus tardivement (en moyenne au bout 51,5 mois de traitement) dans notre série comparée à celle de Lubega et al où 9,5% d'enfants étaient en seconde ligne après 48 mois de traitement [16] ou à celle de Boulle et al qui observaient un taux de passage en seconde de ligne de 24% la même année [21]. Parmi les 39 enfants en échec thérapeutique, 73,4% sont passés au traitement de seconde ligne et 26,6% après l’éducation thérapeutique renforcée ont été maintenus en première ligne de traitement. Mais dans la série de M.-A. Davies et col seulement 50% des enfants en échec étaient passés au traitement de seconde ligne à cause de l'inaccessibilité financière des familles. Dans notre étude, 54% recevaient les combinaisons à dose fixe (comprimés dispersibles) et 46% sous forme de trois médicaments séparés (sirop et/ou comprimés). Plus de la moitié (52,1%) des enfants qui recevaient trois médicaments séparés étaient en échec. L’échec thérapeutique était lié à la complexité du schéma thérapeutique (la forme galénique, le nombre de comprimé, l'association de comprimé et de sirop). L'observance thérapeutique était faible parmi les enfants en échec, 41% (16/39) des familles sont régulièrement venues s'approvisionner en ARV dans les délais durant les 12 premiers mois. Taux deux fois inférieur à celui des parents d'enfants non en échec thérapeutique. La durée moyenne de l'interruption volontaire du traitement était de 11,7 mois. Ce chiffre pourrait expliquer à lui seul la survenue des résistances aux ARV et par conséquence l’échec thérapeutique.

## Conclusion

L’échec thérapeutique était associé à la faible implication des parents à venir chercher le traitement de leur enfant dans les délais, à la mauvaise observance thérapeutique, à une longue durée de l'exposition au traitement ARV et au contexte familial. La charge virale devrait être accessible et disponible pour améliorer la prise en charge des enfants infectés par le VIH et faciliter le passage précoce des enfants en échec thérapeutique en deuxième ligne de traitement en l'absence des traitements de troisième ligne dans notre pays.
